# Three Body-Worn Accelerometers in the French NutriNet-Santé Cohort: Feasibility and Acceptability Study

**DOI:** 10.2196/76167

**Published:** 2025-11-20

**Authors:** Abdouramane Soumaré, Léopold K Fezeu, Jérôme Bouchan, Fabienne Delestre, Alice Bellicha, Greet Cardon, Alan Donnelly, Antje Hebestreit, Mathilde Touvier, Jean-Michel Oppert, Jérémy Vanhelst

**Affiliations:** 1 Université Sorbonne Paris Nord and Université Paris Cité, INSERM, INRAE, CNAM, Centre for Research in Epidemiology and StatisticS (CRESS), Nutritional Epidemiology Research Team (EREN), F-93017 Bobigny, Paris France; 2 Department of Nutrition, Human Nutrition Research Center Ile-de-France (CRNH IdF), Pitié-Salpêtrière Hospital (AP-HP), Sorbonne University Paris, null France; 3 Department of Movement and Sports Sciences, Ghent University Ghent Belgium; 4 Health Research Institute, University of Limerick Limerick Ireland; 5 Leibniz Institute for Prevention Research and Epidemiology-BIPS Bremen Germany

**Keywords:** accelerometer, acceptability, feasibility, cohort study

## Abstract

**Background:**

Accurate assessment of physical activity (PA) in large population-based cohorts remains a major methodological challenge. Self-reported questionnaires, although commonly used due to low cost and simplicity, are prone to recall and social desirability biases, causing misclassification and weakened associations with health outcomes. Body-worn accelerometers provide more objective and reliable measurements, but their acceptability and feasibility in large-scale epidemiological studies must be carefully evaluated to ensure compliance, data quality, and scalability.

**Objective:**

The primary objective was to assess the acceptability of using 3 body-worn accelerometer devices (Fitbit, ActivPAL, and ActiGraph) among healthy middle-aged adults participating in the NutriNet-Santé cohort. The secondary objective was to assess the feasibility of these devices in terms of wear-time compliance under free-living conditions.

**Methods:**

This is an ancillary study of the European WEALTH (WEarable sensor Assessment of physicaL and eaTing beHaviors) project that was conducted between 2023 and 2024 in a subsample of participants of the NutriNet-Santé surveillance in France. This sample included 126 healthy participants (62 men), with a mean age of 46.3 (SD 11.3) years. Participants wore simultaneously 3 body-worn accelerometer devices (Fitbit [wrist], ActivPAL [thigh], and ActiGraph [waist]) for 7 consecutive days. After the wear period, participants completed a specific 22-item web-based questionnaire, regarding their acceptability of using each device. This questionnaire was based on the Technology Acceptance Model, which identifies perceived usefulness and ease of use as key determinants of technology acceptance. Items were rated on a 5-point Likert scale (1=strongly disagree to 5=strongly agree). Feasibility was assessed based on the accelerometer wear time data reported in a log diary by participants. A valid day was defined as ≥600 minutes per day of wear time, and a valid week as at least 4 of such days. Acceptability scores were compared between devices using ANOVA, and feasibility outcomes were compared using Kruskal-Wallis tests.

**Results:**

The acceptability assessment based on the questionnaire revealed significant differences among the 3 devices (*P*<.001). The Fitbit achieved the highest acceptability score (mean 80.5/100, SD 8.13) across most criteria such as comfort, ease of use, and social acceptability, while the ActiGraph received the lowest score (mean 71.7, SD 8.68), mainly due to challenges with stability and interference during PA. In terms of feasibility, the 3 accelerometers demonstrated high compliance, with the ActivPAL recording the highest daily wear time, followed by the Fitbit and the ActiGraph (*P*<.001).

**Conclusions:**

Results from our study showed that the Fitbit watch appears as the most accepted device for measuring PA in free-living conditions in the NutriNet-Santé study. The large-scale use of such a device must now be evaluated in terms of logistics, cost, and data privacy.

## Introduction

Physical activity (PA) is defined as any bodily movement produced by skeletal muscles that results in an energy expenditure higher than the resting state [1]. Robust and consistent evidence supports that PA is a key determinant of health throughout the lifetime [2]. PA is known to improve health and reduce the risk of a wide variety of chronic diseases such as cardiometabolic and respiratory diseases, obesity, and some cancers [3]. Conversely, physical inactivity, defined as not meeting public health PA guidelines, is recognized as the fourth leading risk factor for mortality worldwide [4].

In large epidemiological studies, measuring PA accurately and reliably remains a challenge. Questionnaires are the most commonly used tool in epidemiological studies because of their limited cost and burden (for short questionnaires) and the fact that they are relatively easy to administer on a large scale (especially in web cohorts) [5]. However, self-reported PA methodologies have significant limitations, including recall and social desirability bias, which can lead to meaningful errors in estimating the duration and intensity of PA or to attenuated associations with health end points [6,7]. By comparison, body-worn accelerometers devices measure body segment accelerations (such as wrist, arm, waist, thigh, or ankle) and quantify PA in terms of gravity units or activity counts accumulated over time [8,9]. As such, wearable sensors provide data with a high degree of transparency and harmonization, allowing for comparability across different studies, populations, and countries. High-resolution accelerometry data allow for complex analysis, such as development of PA patterns and performing compositional 24-hour activity analyses in order to associate with health status, to monitor trends and to refine PA guidelines. Hence, accelerometry is considered a more accurate and reliable method for assessing PA in free-living conditions [10,11].

Many accelerometers are available in the health research domain. The most widely used are the research-grade ActiGraph (now branded as Ametris) and the ActivPAL, both devices are found to be reliable and valid for measuring PA and sedentary behavior [12-14]. Recently, a new generation of devices such as the consumer-grade Fitbit has emerged, showing a high popularity among the population due to their user-friendly handling, lower cost, and ability to track multiple metrics in real time [14-16]. Investigators must consider multiple factors to increase participant compliance in wearing the device. While validity and reliability are major factors, the acceptability and feasibility of accelerometer use are also key considerations in determining their potential use in large-scale cohort studies. By definition, acceptability refers to participants’ willingness to wear and use the device [17]. This can be influenced by several parameters such as comfort, ease of use, perceived usefulness, and social acceptability [18]. It is crucial to understand the participants’ preferences, barriers, and facilitators of using body-worn sensors for ensuring long and seamless wear time of devices. Feasibility evaluates the practicality of using wearable devices in free-living conditions. It encompasses several dimensions, including the cost of the devices, the difficulty of ensuring proper mounting, and the logistical requirements, such as meeting participants or sending instructions to explain how to use the device. While these factors are important, the main criterion considered is compliance, specifically the time recorded when the participant effectively wore the device. Indeed, this aspect is essential for obtaining valid and reliable data over long periods, which is necessary for observational studies.

Currently, there is no study assessing the acceptability of wearable devices in middle-aged adults (aged 40-60 years) without chronic diseases. Most studies on the acceptability of PA measurement devices have been conducted using a mixed methods approach and on specific populations such as the older adults [10,19], people with chronic diseases [20], or children and adolescents [21,22]. Middle-aged adults represent an important target group for PA promotion. Studies have shown that increasing PA versus maintaining PA during middle age was associated with comparable lower mortality risk [23]. Moreover, middle-aged adults account for a large proportion of participants in longitudinal cohort studies, making their adherence to monitoring protocols essential for ensuring valid and scalable accelerometer-based surveillance. They thus represent a key target population for future large-scale accelerometer-based analyses. Therefore, the aim of this study was to assess the acceptability and feasibility of using 3 body-worn accelerometer devices in healthy middle-aged adults from the French NutriNet-Santé cohort.

## Methods

### Study Design

This study is an ancillary part of the European WEALTH (WEarable sensor Assessment of physicaL and eaTing beHaviors) project, which generally aims to develop standardized methods for classifying daily behaviors, including PA, from accelerometry and other sensor devices using advanced methods [24]. This study used data collected in France among participants of the NutriNet-Santé study. NutriNet-Santé is a web-based cohort launched in 2009 with the aim to investigate the associations between nutrition and health. As previously described elsewhere, participants older than18 years with access to the web have been continuously recruited since 2009 among the general French population [25]. Individuals reporting chronic diseases, pregnant women, and participants who withdrew their consent before or during data collection did not fulfill eligibility criteria.

Eligibility criteria were (1) a registered participant of the NutriNet-Santé cohort, (2) aged 18 years and older, (3) living in and around Paris (Ile-de-France), (4) be familiar with smartphone technologies, (5) able to understand and communicate in French, and (6) able to attend the laboratory visit for collecting labeled data and to complete data collection during the following 9 days (no holidays, hospital visits, shift work, etc). Among the eligible population, 1350 NutriNet-Santé participants were randomly selected after stratification by sex and age and invited to participate in the study, resulting in a final sample of 156 participants.

### Ethical Considerations

This ancillary study is classified as research involving human person (RIPH2), interventional, except for health products, with minimal risk and constraints in accordance with Article L 1121-1 of the French Public Health Code. The WEALTH study and NutriNet-Santé study were approved by the French ethic committee, conducted in accordance with the Declaration of Helsinki, and approved by the institutional review board of the French Institute for Health and Medical Research (IRB INSERM #0000388FWA00005831) and by the National Commission on Informatics and Liberty (CNIL #908,450 and #909,216). All participants were fully informed about the study’s aims and procedures. Written informed consent was obtained from all participants prior to inclusion. All data collected were pseudonymized, and no participant could be identified in either the analyses or the reporting. Participants did not receive financial compensation. However, they were provided with detailed feedback on their PA behavior and emotional status as a form of nonmonetary incentive. No identifiable images of participants are presented in this manuscript or in supplementary materials.

### Procedures

During the WEALTH study, all French participants were invited to the Pitié Salpêtrière Hospital (APHP, Paris) for an inclusion visit (2 hours) (day 1). During this session, investigators explained the appropriate use of the wearable devices to each participant. Data collection followed a standardized 9-day free-living protocol, consisting of 2 lead-in days (day 1: laboratory visit; day 0: familiarization day), followed by 7 full monitoring days (day 1 to day 7), covering both weekdays and weekend days. Our protocol did not specifically require a valid weekend day, as the primary aim was not to assess detailed PA patterns. Since the Fitbit is widely used in consumer settings, and ActiGraph and ActivPAL are established research-grade devices frequently used in large-scale studies such as National Health and Nutrition Examination Survey, we decided to use these 3 devices in our study. The Fitbit watch (Charge5) was worn on the nondominant wrist, the ActiGraph, now branded as Ametris (wGT3X-BT) on the right waist with an elastic belt, and the ActivPAL (ActivPAL l 3 micro, PAL Technologies Ltd) on the right thigh after being waterproofed in a nitrile sleeve. Participants also wore a LifeQ-enabled smartwatch (Skagen Designs Generation 6) on dominant wrist. However, data from this device were not included in the present analysis. Participants were instructed to wear the ActivPAL continuously, including during sleep, and to remove it only for water-based activities (eg, swimming). In contrast, the ActiGraph was worn only during waking hours and was removed at night, as well as for water-based activities and contact sports. The Fitbit was worn continuously. Prior to the measurement period, participants received an introduction, by investigators, to the wearable devices in a face-to-face session and they could ask any questions. In addition, each participant also obtained written handling manuals to assist with the use and mounting of wearable devices. Finally, participants had an access to a study center hotline for support throughout the wear period. In addition, participants were instructed to keep a log diary collecting when and why the devices (Fitbit, ActiGraph, and ActivPAL) were removed to assess wear and nonwear times. They were also informed about the specific purpose of each wearable device and the rationale to use these different devices simultaneously. The devices were collected after 7-day monitoring period, and data were transferred to a computer for analysis.

### Measurements

#### Participants’ Characteristics

Anthropometric measurements were taken during the inclusion visit at Pitié Salpétrière hospital. Weight and height were measured in light clothes, without shoes, using an electronic scale and a wall-mounted scale, respectively. The BMI was calculated as weight (kg) divided by height² (m^2^). Nutritional status was assessed according to the World Health Organization definitions: underweight (<18.5 kg/m²), normal weight (≥18.5 kg/m² and <25 kg/m²), overweight (≥25.0 kg/m² and <30 kg/m²), and obesity (≥30.0 kg/m²).

#### Acceptability Questionnaire

After the 7-day wear period, participants completed a specific web-based questionnaire regarding their acceptance of using the 3 wearable devices. The 22-item acceptance questionnaire was adapted from previous studies [19,20] and developed within the framework of the Technology Acceptance Model (TAM) [26]. According to TAM, technology adoption is driven by perceived usefulness, perceived ease of use, attitude toward use, and behavioral intention to use. These constructs were operationalized in our questionnaire through specific dimensions: (1) comfort, constraints, and interferences in daily activities (perceived ease of use); (2*i*) changes in PA habits, technical aspects, and perceived usefulness; (3) social acceptability (attitude toward using); and (4) satisfaction and long-term use intention (behavioral intention to use). Participants rated each item on a 5-point Likert scale, indicating the extent to which they agreed with each statement, from 1 (strongly disagree) to 5 (strongly agree). In addition, participants completed a ranking of the devices on their preferences for wearing them in future studies and also provided open-ended feedback on their experience with PA measurement technologies. The questionnaire is available on the web (in French) [27].

#### Feasibility

Feasibility was assessed through participants’ compliance with wearing the body-worn accelerometer devices, using data from the log diary. Participants were asked to record the time of all device removals and replacements during the consecutive 7-day period. Wear time for the 3 devices was calculated for each day by summing the durations noted in the log diary. Established accelerometer wear time criteria were used to calculate the number of valid days (defined as a day with wear time ≥600 minutes) for each device, as well as the number of valid weeks (defined as a week with at least 4 valid days) [12]. These 2 thresholds are widely used in accelerometer-based studies among adults [12]. Indeed, in a systematic review with practical considerations, the authors recommended a minimum of 600 minutes per day (10 hours) and at least 4 valid days per week to ensure that the accelerometer captures a representative portion of daily activity and provides reliable estimates of habitual PA patterns under free-living conditions [12].

### Statistical Analysis

For the description of participants’ characteristics, means and standard deviations were calculated for continuous variables, and number and percentage for categorical variables. For the acceptance questionnaire assessing the use of the activity tracker, the average score for each item was calculated. Total acceptability scores for each device were obtained by summing the scores from individual items and were then compared using an ANOVA test with a significance level of *P*<.05. After checking for normality, the daily wear time of the 3 body-worn accelerometer devices (in minutes per day) was described using medians and the 25th and 75th percentiles. The median wear time for these 3 devices over the 7-day period was calculated and compared using a Kruskal-Wallis test, with a significance level of *P*<.05. All analyses were performed in SAS software (version 9.4; SAS Institute).

## Results

### Characteristics of Participants

Among the 156 participants, 150 had valid data on wear time for all 3 wearable devices. Of these, 130 completed the acceptance questionnaire, with 126 providing complete data for analysis. The characteristics of the included participants are described in Table 1. The study sample consisted of 126 adults (62 men, 64 women), with a mean average age of 46.3 (SD 11.3) years. In addition, 52.4% (66/126) of participants reported having prior experience with body-worn accelerometer devices.

**Table 1:** Characteristic of participants (N=126).

Characteristics			Participants, n (%)		Mean (SD)
**Gender**					
	Men	62 (49.2)		N/A^a^	
	Women	64 (50.8)		N/A	
	Age (years)	N/A		46.3 (11.3)	
	Height (cm)	N/A		170 (10.1)	
	Weight (kg)	N/A		69.7 (12.5)	
	BMI (kg/m²)	N/A		23.9 (3.19)	
**BMI categories**					
	Underweight	1 (0.79)		N/A	
	Normal	85 (67.5)		N/A	
	Overweight	35 (27.8)		N/A	
	Obesity	5 (4)		N/A	
**Experience using PA^b^ measurement technologies**					
	Yes	66 (52.4)		N/A	
	No	60 (47.6)		N/A	

^a^N/A: not applicable.

^b^PA: physical activity.

### Acceptability

Evaluation of the 3 devices using the acceptability questionnaire revealed a significant overall difference in scores (*P*<.001). Pairwise comparisons also showed differences, with the Fitbit obtaining the highest score (mean 80.5, SD 8.13 points) and the ActiGraph (mean 71.7, SD 8.68) the lowest (Table 2).

However, there was variability in scores according to the different concepts measured in the acceptance questionnaire (Table 3). The Fitbit watch was perceived as the most comfortable to wear overall, followed by the ActivPAL and then the ActiGraph, despite the ActiGraph obtaining the lowest scores for items related to skin conditions (items 2 and 3). For these same items, the ActiGraph scored higher than the other devices, indicating that it caused fewer skin irritations.

Regarding the items related to constraints and interference with daily activities, all 3 devices obtained satisfying scores (above 3.6/5), although scores were slightly lower for interference with sleep (item 6). The ActiGraph had the lowest scores on these criteria (items 5-8). Changes in PA habits (item 9) were most pronounced for the Fitbit watch, which obtained the lowest score (3.71/5). All 3 devices received relatively high scores (4.32/5) for ease of use (item 10). However, the ActivPAL and the ActiGraph had lower scores (2.70 and 2.65, respectively) than the Fitbit (4.06/5) for understanding the device-wearing instructions (item 11).

Regarding social acceptability (items 14-17), the Fitbit watch obtained higher scores than the other devices, while the ActiGraph had the lowest scores, particularly for discretion (2.79) and esthetics (2.32). All 3 devices scored well on items related to overall satisfaction and intention to use in the long term in the NutriNet Santé study (items 18 and 19), with the Fitbit watch and the ActivPAL showing the highest scores.

**Table 2:** Average acceptability score obtained by participants for each accelerometer.

	Mean (SD) score	Minimum-Maximum	*P* value^a^
Fitbit	80.5 (8.13)	59-95	N/A^b^
ActivPAL	77.8 (8.87)	50-95	.015
ActiGraph	71.7 (8.68)	45-89	<.001

^a^Reference: Fitbit. Minimum score: 19; maximum score: 95. Global *P* value for ANOVA: *P*<.001.

^b^N/A: not applicable.

**Table 3:** Results (answer averages) of the acceptance questionnaire assessing the use of wearable accelerometersa.

Item			Fitbit		ActivPAL		ActiGraph		Average
**Comfort level**									
	1. In general, I found “the device” comfortable to wear.	4.11		3.89		3.10		3.7	
	2. “The device” irritated my skin (itching, redness, unpleasant sensation, etc).^b^	4.01		4.06		4.67		4.25	
	3. “The device” left traces on my skin (Marks, visible lines).^b^	3.95		3.81		4.66		4.14	
	4. After a few hours, I was still aware that I was wearing “the device.”^b^	3.75		4.00		2.98		3.58	
**Constraints and interferences in daily activities**									
	5. I found it easy to work while wearing the “device.”	4.70		4.77		4.42		4.63	
	6. I found it hard to sleep while wearing the “device.”^b^	3.72		3.99		3.24		3.65	
	7. I found it easy to carry out my daily activities (leisure, housework) while wearing “the device.”	4.75		4.68		4.27		4.57	
	8. I found it easy to put on my usual clothes while wearing “the device.”	4.83		4.41		3.56		4.27	
**Changes in PA^c^ habits**									
	9. Wearing the “device” made me move more than usual.^b^	3.71		4.12		4.13		3.99	
**Ease of use**									
	10. Overall, I found “the device” easy to use.	4.37		4.47		4.12		4.32	
	11. The instructions and guidelines provided by the researcher on the use of the “device” were difficult to understand.^b^	4.06		2.70		2.65		3.14	
**Technical reports**									
	12. Overall, I was satisfied with the autonomy of the “device.”	4.33		4.69		4.36		4.46	
	13. I found the charging time of the “device” too long.^b^	4.10		4.53		4.52		4.38	
**Social acceptability**									
	14. I found the “device” esthetically good.	3.81		2.72		2.32		2.95	
	15. I found the size of the “device” to be appropriate and discreet.	4.11		3.79		2.79		3.56	
	16. I found it embarrassing to wear “the device” in public.^b^	4.5		3.93		3.40		3.94	
	17. I have had some negative comments from other people when I was wearing “the device.” ^b^	4.84		4.78		4.63		4.75	
**Satisfaction and long-term use intention**									
	18. Overall, I’m disappointed with my experience with the “device.”^b^	4.29		4.05		3.73		4.02	
	19. If I were asked to wear the “'device”' for a week within the NutriNet Santé study, I would definitely accept.	4.52		4.44		4.19		4.38	
Average by device, mean (SD)			4.23 (0.43)		3.78 (0.46)		4.10 (0.47)		N/A^d^

^a^Scoring: 1=Strongly disagree, 2=disagree, 3=Neither agree nor disagree, 4=Agree, and 5=Strongly agree.

^b^Items were negatively framed and therefore reverse-coded prior to analysis.

^c^PA: physical activity.

^d^N/A: not applicable.

Table 4 summarizes the positive and prohibitive negative points most frequently cited by participants for each accelerometer. For the Fitbit watch, which had the highest acceptability score, the most frequently mentioned positive aspect was its diverse functionalities (sleep tracking, physical behavior, and other parameters). In contrast, for the ActiGraph, the device with the lowest score, the most recurrent noted negative point was its instability during wear, particularly during sports activities.

**Table 4:** Most recurrent positive and negative statements for each device (N=126).

		Most recurrent statement	Participants, n (%)
**Fitbit**			
	Positive statement	Tracks activity, sleep, and other parameters (heart rate, number of steps, and notifications)	47 (37.6)
	Negative statement	Skin affections (irritation, itching, marks, allergies, plastic, discomfort...)	37 (29.6)
**ActivPAL**			
	Positive statement	Discreet and comfortable (easy to forget, lightweight, invisible...)	78 (62.4)
	Negative statement	Skin affections (irritation, redness, itching, and marks after removal)	20 (16)
**ActiGraph**			
	Positive statement	Easy to use (easy to remove, replace, attach to belt, and adjustable)	33 (26.4)
	Negative statement	Unstable (difficult to hold in place, elastic waistband slips, and moves during certain sports activities)	48 (38.4)

### Feasibility

The description and comparison of the wear time for each device (medians and 25th and 75th centiles) over the 7-day wear period are shown in Table 5. A significant difference was found among the devices. Comparative analysis showed that the ActivPAL had the greatest wear time, with a significant difference compared with both ActiGraph (*P*<.001) and Fitbit (*P*<.001). Pairwise comparisons also showed differences between ActivPAL and Fitbit (*P*<.001). The Fitbit also exhibited a higher wear time than the ActiGraph (*P*<.001). Overall, the ActivPAL had a total of 1049 valid days (person × day), the Fitbit accumulated 1046 valid days (person × day), and the ActiGraph had 1006 valid days (person × day). All participants validated the week of PA evaluation, that is, achieving at least 4 valid days with a wear time of ≥600 minutes per day.

**Table 5:** Wear time (minutes per day) for the 3 accelerometers.

	Median wear time (25th; 75th centile)				*P* value
	ActiGraph	ActivPAL	Fitbit		
Day 1	871 (795; 941)	1440 (1440; 1440)	1440 (1390; 1440)		
Day 2	930 (885; 983)	1440 (1440; 1440)	1440 (1440; 1440)		
Day 3	950 (887; 1050)	1440 (1440; 1440)	1440 (1415; 1440)		
Day 4	945 (855; 1340)	1440 (1440; 1440)	1440 (1435; 1440)		
Day 5	975 (899; 1335)	1440 (1440; 1440)	1440 (1440; 1440)		
Day 6	964 (895; 1057)	1440 (1440; 1440)	1440 (1440; 1440)		
Day 7	890 (808; 954)	1440 (1440; 1440)	1440 (1440; 1440)		
Over the 7 days	930 (855; 1020)^a,b^	1440 (1440; 1440)^b,c^	1440 (1421; 1440)^a,c^	<.001	

^a^Pairwise post hoc comparisons (ActivPAL).

^b^Pairwise post hoc comparisons (Fitbit).

^c^Pairwise post hoc comparisons (ActiGraph).

## Discussion

### Principal Findings

There is a pressing need to improve methods for assessing PA in large cohort studies. An important challenge is to identify the most suitable device by finding the optimal compromise between acceptability and feasibility for the participants. The aim of our study was to assess the feasibility and acceptability of using body-worn accelerometer devices (ActiGraph, ActivPAL, and the Fitbit watch) in a subsample of the NutriNet-Santé cohort for potential future implementation. In terms of acceptability and feasibility, our study suggests that the Fitbit would be the most suitable device for measuring PA in free-living conditions in the framework of a large cohort such as the NutriNet-Santé study.

The 3 devices were perceived differently on the different dimensions of acceptability. The Fitbit watch and ActivPAL were rated the most comfortable overall, despite receiving lower scores for skin-related issues, whereas the ActiGraph was evaluated more positively in terms of skin conditions. The Fitbit watch was worn on the wrist, a familiar placement for such devices, which could explain why participants perceived it as the most comfortable. However, like the ActivPAL, it requires direct skin contact, which can cause irritation or marks, a concern not shared by the ActiGraph, which can be worn over clothing. The most frequently reported negative aspect by participants regarding the Fitbit watch is that it causes irritations and marks. These conditions may be due to the plastic or to specific materials of the wristband.

In terms of interference with daily activities, the high scores for all 3 devices indicate that they cause minimal disruption to participants’ daily movement and life. However, participants rated the ActiGraph lower score due to interference with wearing regular clothes, possibly due to the need for a fastening belt, which may not be suitable for all types of clothing. The impact on sleep may reflect the comfort level for the ActiGraph and the ActivPAL devices, as well as the notifications and light signals emitted by the Fitbit watch, which may disturb rest. Comfort level and interference with daily activities are key aspects that may influence participants’ attitudes toward the devices (“Attitude Toward Using”) and impact their intention for future use, as explained by the TAM.

### Comparison With Previous Work

Although notifications were sent only to participants’ phones as part of this protocol, the Fitbit watch received a valuable score for change in PA habits, which actually reflects its potential to influence behavior through real-time tracking and feedback that may encourage PA changes. Indeed, these features correspond to the most frequently cited positive aspect by participants about the Fitbit watch, reflecting its Perceived Usefulness, 1 of the 2 main determinants of acceptability according in the TAM [26]. Similar findings have been observed in a study performed in adolescents, where devices with interactive features positively impacted PA behavior [28]. However, there is no evidence supporting that this reactivity persists over time. A study on the reactivity of a pedometer with interactive feedback features worn for 2 weeks demonstrated that this reactivity lasts only about a week, and in the absence of intervention, habitual behaviors return during the second week, representing a more accurate estimate of habitual PA in adults [29]. Therefore, we recommend measuring daily PA levels after 1 week of wearing such a device, using the first week as a priming phase. A second option is to consider a blinding procedure when feasible.

All 3 devices were perceived positively in terms of ease of use, although participants found the instructions for the ActivPAL and the ActiGraph more complicated to understand. This suggests that the clarity of the instructions could be improved to enhance Perceived Ease of Use, another key determinant of acceptability in the TAM [26]. In terms of social acceptability, the Fitbit watch exhibited the highest score, while the ActiGraph and the ActivPAL were noted less favorably, with the lowest scores for the ActiGraph, particularly in terms of esthetics, discretion, and wearing in public. The differences observed between the ActiGraph and the ActivPAL lie in the fact that the ActivPAL tends to remain invisible under clothing during the winter season, unlike the ActiGraph, which is hard to miss if worn over clothing. These negatively judged esthetic aspects may lead to a negative attitude of participants toward the devices “Attitude Toward Using,” which would unfavorably affect the intention for long-term use, according to the TAM [26].

Concerning feasibility, the results of our study showed that the ActivPAL accelerometer had the highest compliance level, which is not surprising given that it is attached with waterproofed and adhesive dressing to the skin of the thigh. This was followed by the Fitbit watch and the ActiGraph. A review also highlighted that the ActivPAL demonstrated longer wear times than the ActiGraph, potentially due to differences in wear protocols and attachment methods [30]. Indeed, the lower ranking of the ActiGraph can be explained by its placement around the waist and its removal for certain daily activities, such as contact sports, high-impact activities, and water-based activities (swimming, showering, bathing, etc) due to its nonwater resistance. Participants were also asked to remove this device during sleep periods (from bedtime to waking time), which could lead to a risk of forgetting to put it back on, thus affecting the data quality [31]. The Fitbit watch has a high compliance level compared with the ActiGraph, likely due to its wrist placement and water resistance. Indeed, a study has shown that compliance is higher for accelerometers worn on the wrist than on the waist [10]. However, the Fitbit watch needs to be removed at least once for recharging during a 1-week assessment of daily PA. This might explain the lower compliance of this device compared with the ActivPAL, which has a battery life of more than 7 days. Wearing an accelerometer on the wrist and ensuring that it is waterproof enhance continuous use both day and night, improving overall compliance [31,32]. In 2011, the National Health and Nutrition Examination Survey study (United States) using the ActiGraph accelerometer modified the data collection protocol, transitioning from waist-worn to wrist-worn to improve participant compliance and add sleep data [32]. This change aligns with the increasing importance of recent studies investigating 24-hour movement (sedentary lifestyle, PA, and sleep) and their combined effects on health [33-35]. Several studies suggest that health outcomes are influenced by the balance among these 3 behaviors [33-37]. These findings highlight the importance of technical characteristics such as battery life, waterproofing, and placement for ensuring compliance in epidemiological studies. However, to reproduce such a protocol in a large cohort remains a challenge. Indeed, despite the higher compliance found for the ActivPAL, this device requires in-person visits for proper placement, as it is attached to the skin using adhesive dressing, and its removal or replacement is more complex compared with other devices. These logistical and technical constraints contrast with the ActiGraph and the Fitbit, which can be easily mailed to participants with instructions and self-applied, making it more practical for large-scale studies. Nonetheless, the observed compliance level for the 3 accelerometers is excellent, as they have only valid weeks (4 days with wear time ≥600 minutes per day), which is required to obtain valid measurements of PA in free-living conditions, as previously also reported in a review between ActiGraph and ActivPAL [30].

In summary, the results from our study indicate that the Fitbit watch is the most accepted body-worn accelerometer device by participants compared with the ActiGraph and the ActivPAL. Its lower cost (€159.95 [US $174.20]) compared with the other 2 devices (>€292 [US $318]) also makes it a favorable option for use in large-scale population studies. Thus, this tool represents the best compromise between acceptability and feasibility and may be a suitable alternative for a large web cohort, such as the NutriNet-Santé study. In addition, previous models of the Fitbit watch have shown acceptable validity for assessing moderate to vigorous intensity PA (*r*=0.73 compared with ActiGraph GT3X+) [38], sedentary behavior (intraclass correlation coefficient=0.95% compared with ActivPAL) [39], total daily energy expenditure (*r*=0.84 compared with doubly labeled water in free-living conditions) [40], and sleep (sensitivity=0.96; specificity=0.61 compared with polysomnography) [41].

### Limitations

Our study has several limitations that should be acknowledged. First, wearing 4 devices simultaneously could have caused discomfort and make it difficult for participants to assess each sensor individually. Second, the study was conducted between September and February. Seasonal variations, particularly during summer, may affect participants’ comfort and the discretion of devices, such as the ActivPAL, which is typically worn under clothing. Third, although the ActiGraph is now widely worn on the wrist in research studies, we were unable to assess this placement, as participants were already wearing 2 wrist-based accelerometers (Fitbit and LifeQ), which may have influenced perceptions and comparability. Indeed, we could consider that the position of the device, rather than the device itself, may be a critical factor influencing our results [42]. Fourth, since participation in the NutriNet-Santé cohort is voluntary, individuals tend to be healthier and have higher education levels than the general French population. In addition, participants likely adhered better to the study methodology because they are active cohort involvement, which may lead to higher acceptance. Therefore, our results cannot be extrapolated to all populations. Fifth, the apparent low recruitment rate (11.6%) should not be interpreted as a recruitment difficulty but rather as a direct consequence of the original study design. In the WEALTH project, it had been decided to include only 150 participants per country according to the initial sample size calculation. Therefore, in our ancillary study, that target was met and it was not possible to include more participants. Other factors should also be considered including the validity and reliability of data collected from these different wearable devices, that could affect the findings. Finally, we acknowledge that our study did not assess the quality, integrity, or stability of the data collected by the 3 devices. Future studies should specifically address these aspects to complement our findings.

### Conclusions

Our study demonstrates that body-worn accelerometers are both feasible and acceptable among middle-aged adults in a large French web-based cohort. The Fitbit watch appears the most accepted device to measure PA in free-living conditions in participants. The Fitbit is also commercially available, which could facilitate broader participation by allowing individuals to contribute data from their personal devices. This accessibility could have a positive impact on the scalability of PA measurements in epidemiological studies. In addition, this type of device may be suitable to measure PA over several weeks or months at different times throughout the year, providing valuable insights into seasonal variations in PA. However, it is crucial to address data privacy concerns to protect participants’ personal information and ensure the accuracy of the PA data collected and analyzed by researchers.

## Abbreviations

PA: physical activity
TAM: Technology Acceptance Model
WEALTH: WEarable sensor Assessment of physicaL and eaTing beHaviors

## Data Availability

The datasets used and/or analyzed during this study are available from the corresponding author on reasonable request. Researchers from public institutions can submit a collaboration request including their institution and a brief description of the project to collaboration@etude-nutrinet-sante. All requests will be reviewed by the steering committee of the NutriNet-Santé study. A financial contribution may be requested. If the collaboration is accepted, a data access agreement will be necessary and appropriate authorizations from the competent administrative authorities may be needed. In accordance with existing regulations, no personal data will be accessible.
